# Effect of acute hypoxia on respiratory muscle fatigue in healthy humans

**DOI:** 10.1186/1465-9921-11-109

**Published:** 2010-08-11

**Authors:** Samuel Verges, Damien Bachasson, Bernard Wuyam

**Affiliations:** 1 HP2 laboratory (INSERM ERI17), Joseph Fourier University and Exercise Research Unit, Grenoble University Hospital, Grenoble (38000), France

## Abstract

**Background:**

Greater diaphragm fatigue has been reported after hypoxic versus normoxic exercise, but whether this is due to increased ventilation and therefore work of breathing or reduced blood oxygenation per se remains unclear. Hence, we assessed the effect of different blood oxygenation level on isolated hyperpnoea-induced inspiratory and expiratory muscle fatigue.

**Methods:**

Twelve healthy males performed three 15-min isocapnic hyperpnoea tests (85% of maximum voluntary ventilation with controlled breathing pattern) in normoxic, hypoxic (SpO_2 _= 80%) and hyperoxic (FiO_2 _= 0.60) conditions, in a random order. Before, immediately after and 30 min after hyperpnoea, transdiaphragmatic pressure (P_di,tw _) was measured during cervical magnetic stimulation to assess diaphragm contractility, and gastric pressure (P_ga,tw _) was measured during thoracic magnetic stimulation to assess abdominal muscle contractility. Two-way analysis of variance (time x condition) was used to compare hyperpnoea-induced respiratory muscle fatigue between conditions.

**Results:**

Hypoxia enhanced hyperpnoea-induced P_di,tw _and P_ga,tw _reductions both immediately after hyperpnoea (P_di,tw _: normoxia -22 ± 7% vs hypoxia -34 ± 8% vs hyperoxia -21 ± 8%; P_ga,tw _: normoxia -17 ± 7% vs hypoxia -26 ± 10% vs hyperoxia -16 ± 11%; all *P *< 0.05) and after 30 min of recovery (P_di,tw _: normoxia -10 ± 7% vs hypoxia -16 ± 8% vs hyperoxia -8 ± 7%; P_ga,tw _: normoxia -13 ± 6% vs hypoxia -21 ± 9% vs hyperoxia -12 ± 12%; all *P *< 0.05). No significant difference in P_di,tw _or P_ga,tw _reductions was observed between normoxic and hyperoxic conditions. Also, heart rate and blood lactate concentration during hyperpnoea were higher in hypoxia compared to normoxia and hyperoxia.

**Conclusions:**

These results demonstrate that hypoxia exacerbates both diaphragm and abdominal muscle fatigability. These results emphasize the potential role of respiratory muscle fatigue in exercise performance limitation under conditions coupling increased work of breathing and reduced O_2 _transport as during exercise in altitude or in hypoxemic patients.

## Introduction

It is well known that acute hypoxia results in a reduction of maximal exercise work rate and endurance performance [1-3]. The mechanisms responsible for this reduction are however complex. It has been suggested that 'central' factors, including pulmonary gas exchange, cardiac output [1] or cerebral perturbations [4] are mainly involved. Whether hypoxia increases peripheral muscle fatigue per se has been a matter of debate [5,6]. Recent results indicate however that a cycling bout of similar workload and duration induced a greater impairment of quadriceps contractility in hypoxia compared to normoxia [7]. In addition to locomotor muscles, it is now recognized that intensive whole-body exercise also induces respiratory muscle fatigue [8-10]. Under hypoxic conditions, exercise-induced diaphragm fatigue was shown to be enhanced compared to normoxia [11-13]. Hypoxia has however multiple effects on the physiological response to whole-body exercise that may interact with locomotor and respiratory muscle fatigue development and other reasons than reduced O_2 _transport to the diaphragm may affect diaphragm fatigue in hypoxia. First hypoxia increased minute ventilation and consequently the work of breathing, therefore potentially leading to greater muscle fatigue. Second, hypoxia might enhanced blood flow competition between respiratory and locomotor muscles [14]. Third, hypoxia can influence the amount of circulating metabolites (e.g. increased lactate) produced in locomotor muscles working at a higher relative intensity compared to normoxia [11].

To assess specifically the effect of hypoxia on muscle fatigue independently of confounding factors associated with whole-body exercise, isolated exercise protocol can be used, together with objective measurements of muscle contractile properties before and after exercise, as obtained from evoked contractions in response to artificial nerve stimulation. Katayama et al [15] recently measured quadriceps twitch force during magnetic femoral nerve stimulation before and after intermittent submaximal isometric quadriceps contractions under normoxic and hypoxic (arterial oxygen saturation, SpO_2 _= 75%) conditions and showed greater fatigability in hypoxia. The effect of hypoxia on muscle fatigue may however depend on the muscle group (differing in fibre types, oxidative capacities and capillarisation) and the type of contraction (e.g. isometric versus dynamic), as recently reviewed by Perrey et al. [16]. Some studies have used inspiratory resistive breathing protocols to evaluate the effect of reducing the inspiratory oxygen fraction (FiO_2 _) on inspiratory muscle endurance and fatigue leading to contrasting results: some results showed reduced inspiratory muscle endurance [17,18], while others indicated similar inspiratory muscle fatigability (assessed by maximal voluntary inspiratory manoeuvres, [19]) in hypoxia compared to normoxia. Inspiratory resistive breathing however induces different type of muscle contraction (high load - low speed) compared to hyperpnoea (low load - high speed, similar to spontaneous breathing during exercise). The effect of hypoxia on hyperpnoea-induced diaphragm fatigue objectively assessed by phrenic nerve stimulation remains therefore to be investigated. Furthermore, expiratory muscles (abdominal muscles mainly) have a critical role during exercise-induced hyperpnoea [20] and can also fatigue during intensive exercise [9,10]. The effect of hypoxia on hyperpnoea-induced abdominal muscle fatigue is however unknown.

In the present study, we aimed to assess the effect of different blood oxygenation level on isolated hyperpnoea-induced inspiratory and expiratory muscle fatigue. We therefore measured diaphragm and abdominal muscle twitch responses to cervical and thoracic magnetic stimulation, respectively, before and after a standardized bout of isocapnic hyperpnoea. We hypothesized that hypoxia would increase and hyperoxia would decrease both inspiratory and expiratory muscle fatigue development during voluntary isocapnic hyperpnoea.

## Materials and methods

### Subjects

Twelve healthy, non-smoking, men were studied. Subjects' characteristics are shown in Table 1. Subjects refrained from physical exercise on the 2 days prior to the tests, refrained from drinking caffeinated beverages on test days, and were required to have their last meal at least 2 h prior to the tests. The study was approved by the local ethics committee (Grenoble, Sud Est V) and performed according to the Declaration of Helsinki. All subjects gave their written informed consent to participate in the study.

**Table 1:** Subjects' characteristics

Age	(yrs)	31.8 (9.5)
Body mass	(kg)	71 (7.5)
Height	(cm)	178 (6)
VC	(l)	5.85 (0.93)
	(% predicted)	111.5 (21.6)
FEV1	(l·s^-1^)	4.66 (0.77)
	(% predicted)	107.7 (6.3)
MVV	(l·min^-1^)	190.6 (37.46)
	(% predicted)	101.4 (14.7)

Values are means (SD). VC, vital capacity; FEV1, forced expiratory volume in one second; MVV, maximum voluntary ventilation.

### Protocol

Subjects performed four test sessions at least 72 h apart. The first session consisted in lung function measurements (Ergocard, Medisoft, Dinant, Belgium) according to standard procedures [21] and familiarization with cervical and thoracic magnetic stimulations. The subjects also performed the 15-min hyperpnoea test at their individual target minute ventilation (see below) to familiarize themselves with this procedure. The next three sessions started with diaphragm and abdominal muscle strength measurements with cervical and thoracic magnetic stimulations, respectively (see below). Then, subjects breathed quietly for 10 min before starting the 15-min hyperpnoea test. Immediately after the end of hyperpnoea as well as after 30 min of recovery with quiet room air breathing (i.e. a time period previously shown to allow only partial recovery of fatigue, [9,22]), diaphragm and abdominal muscle strength measurements were repeated. The 10-min quiet breathing period as well as the 15-min hyperpnoea test were performed i) while breathing room air (FiO_2 _= 21%, laboratory altitude: 200 m, i.e. normoxia), ii) with a SpO_2 _of 80% (i.e. hypoxia) and iii) with a FiO_2 _= 60% (i.e. hyperoxia). The order of normoxia, hypoxia and hyperoxia conditions was randomized over the three test sessions. Subjects were blinded for the inhaled gas mixture. In two subgroups, diaphragm and abdominal muscle strength measurements were also performed after the 10-min quiet breathing period in hypoxia (6 subjects) and hyperoxia (6 subjects), in order to assess the effect of hypoxia and hyperoxia on respiratory muscle contractility at rest.

### Hyperpnoea test

The subject sat comfortably on a chair and breathed on a mouth piece and a three-way valve to an ergospirometric device (Ergocard, Medisoft, Dinant, Belgium). The inspiratory side of the valve was connected to a specific device (prototype SMTEC, Nyon, Switzerland) able to deliver a gas mixture with an O_2 _fraction from 5 to 60% and a carbon dioxide (CO_2 _) fraction from 0 to 6% supplemented with nitrogen at flow rates up to 200 l·min^-1 ^with negligible resistance. O_2 _and CO_2 _fractions could be modified continuously in order to maintain normocapnia (continuously checked by measuring end-tidal partial CO_2 _pressure, P_ET _CO_2 _) and to reach the target SpO_2 _level under hypoxic condition. After 10-min of quiet breathing, the subject had to breathe for 1 min at 60% of maximal voluntary ventilation (MVV) and then for 14 min at 85% MVV, i.e. a ventilatory level leading to similar amount of fatigue than following an exhaustive exercise [11,23]. The subject had a continuous breath by breath feedback regarding minute ventilation and breathing frequency in order to match his target ventilatory level and breathing pattern. FiCO_2 _was set by the experimenter to maintain P_ET _CO_2 _at the same level than during the quiet breathing period. During both the 10-min quiet breathing period and the 15-min hyperpnoea period, FiO_2 _was set at 21% during the normoxic session, at 60% during the hyperoxic session and was adjusted to maintain SpO_2 _= 80% during the hypoxic session. Breath by breath ventilatory variables, SpO_2 _and heart rate (HR) were measured continuously (Ergocard, Medisoft) while subjects' rate of perceived exertion was assessed every 2 min on a visual analogue scale. In 6 subjects, at rest and after 8 min of hyperpnoea (as a representative time point of the total hyperpnoea period) in each conditions (normoxia, hypoxia and hyperoxia), 125 μL and 20 μl arterialized blood samples were drawn from the earlobe and analyzed immediately to determine arterial blood gas, pH (SGI Microzym-L, Toulouse, France) [24] and blood lactate concentration ([La], AVL instruments, Graz, Austria), respectively.

### Magnetic stimulation

Cervical and thoracic magnetic stimulations were performed by using a circular 90-mm coil powered by a Magstim 200 stimulator (MagStim, Whitland, UK) as previously described [23]. Oesophageal (P_oes _) and gastric (P_ga _) pressures were measured by conventional balloon catheters [25], connected separately to differential pressure transducers (model DP45-30; Validyne, Northridge, CA). Transdiaphragmatic pressure (P_di _) was obtained by online subtraction of P_oes _from P_ga _. Pressure analogue signals were digitized (MacLab, ADInstruments, Castle Hill, Australia) and recorded simultaneously on a computer (Chart Software version 5.0; ADInstruments; sampling frequency: 2 kHz). Cervical magnetic stimulation of the phrenic nerves was performed while subjects were seated comfortably in a chair with the centre of the coil positioned at the seventh cervical vertebra [26]. Thoracic stimulation of the nerve roots innervating the abdominal muscles was performed while subjects lay prone on a bed with the centre of the coil positioned at the intervertebral level T10 [27]. The best spot allowing the maximal twitch pressures (P_di,tw _and P_ga,tw _) was determined with minor adjustments and then marked on the skin for the remainder of the experiment. Subject and coil positions were checked carefully throughout the experiment. The order of cervical and thoracic stimulations was randomized between subjects but was the same over all sessions of a given subject. To avoid the confounding effect of potentiation [27,28], subjects performed three 5-s maximal inspiratory efforts from functional residual capacity (for cervical stimulation) or three 5-s maximal expiratory efforts from total lung capacity (for thoracic stimulation) against a closed airway prior to a series of six stimulations at 100% of the stimulator output. After three stimulations, another 5-s maximal voluntary contraction followed. All stimuli were delivered at functional residual capacity after a normal expiration, with the airway occluded. To ensure the same lung volume at all times before and after exercise, the experimenter checked that for each subject pre-stimulation P_oes _ranged at the same level immediately before each cervical or thoracic stimulations. Recordings that showed changes in pre-stimulation P_oes _were rejected post hoc. For data analysis, the average amplitude (baseline to peak) of all remaining twitches (at each stimulation site) was calculated. P_oes,tw _/P_ga,tw _ratio during cervical stimulation was calculated as an index of extra-diaphragmatic inspiratory muscle fatigue [29]. The procedure for P_di,tw _and P_ga,tw _measurement before and after hyperpnoea took 5 to 6 min. Within-day coefficients of variation were 3% for P_di,tw _during cervical stimulation and 4% for P_ga,tw _during thoracic stimulation. Between-day coefficients of variation were 6% for P_di,tw _during cervical stimulation and 9% for P_ga,tw _during thoracic stimulation.

To check for supramaximal stimulation, additional twitches were performed with 80, 90, 95, and 98% of the maximal stimulator output (6 twitches at each stimulator intensity) during cervical and thoracic stimulation at the beginning of each test session. Supramaximality of magnetic stimulation was confirmed by reaching, at submaximal outputs of the stimulator, maximal levels of P_di,tw _during cervical stimulation in all subjects and maximal levels of P_ga,tw _during thoracic stimulation in all subjects but three [23,30]. Since the last three subjects had similar results than the rest of the group (i.e. twitch amplitude reductions in the three conditions), there were included in all analysis.

### Data analysis

All descriptive statistics presented are mean values ± SD. The comparison of parameters between the three conditions (normoxia, hypoxia, and hyperoxia) was achieved using two-way analysis of variance (ANOVA, time x condition) with repeated measurements. When significant main effects were found, Fischer's p-test was used for post hoc analysis. All statistical calculations were performed on standard statistics software (Statview 5.0, SAS Institute, Cary, North Carolina). Significance was set at *P *< 0.05.

## Results

The two main dependent variables in this study were the reduction in P_di,tw _and P_ga,tw _after hyperpnoea under normoxic and hypoxic conditions. The power for P_di,tw _was 100% and for P_ga,tw _it was 99%.

### Ventilation and physiological responses during hyperpnoea

Average ventilation, blood gases, [La], HR and rate of perceived exertion during the 15-min hyperpnoea test in normoxia, hypoxia and hyperoxia are shown in Table 2. Minute ventilation, breathing pattern, P_ET _CO_2 _, PaCO_2 _and pH were not different between the three conditions. SpO_2 _and PaO_2 _were significantly lower in hypoxia compared to normoxia and hyperoxia, while PaO_2 _was significantly higher in hyperoxia compared to normoxia and hypoxia. The target SpO_2 _during hypoxia was adequately maintained over the 15 min of hyperpnoea with a mean coefficient of variation of 3%. The mean FiO_2 _during the 15-min hyperpnoea in hypoxia was 9.7 ± 1.2% (range: 8-11%). [La] was higher in hypoxia compared to hyperoxia (*P *= 0.032) and normoxia (*P *= 0.095). Similarly, HR was higher in hypoxia compared to hyperoxia (*P *= 0.015) and normoxia (*P *= 0.070). Rate of perceived exertion was not significantly different between conditions.

**Table 2:** Average ventilation, blood gases, blood lactate concentration, heart rate and perceived level of exertion during the 15-min hyperpnoea test in normoxia, hypoxia and hyperoxia

	Normoxia	Hypoxia	Hyperoxia
${\overset{•}{\text{V}}}_{E}$ (l min^-1^)	159.3 (15.6)	158.1 (15.6)	159.5 (15.1)
f_R _(cycles·min^-1^)	54.2 (4.3)	54.2 (4.9)	53.7 (4.1)
V_T _(l)	2.95 (0.26)	2.93 (0.30)	2.98 (0.25)
Ti/Tt	0.48 (0.03)	0.49 (0.03)	0.49 (0.04)
P_ET _CO_2 _(mmHg)	37.0 (2.1)	36.8 (1.7)	35.9 (2.3)
SpO_2 _(%)	98.4 (0.9)	79.3 (1.9) ***	98.9 (0.7)
PaO_2 _(mmHg) (n = 6)	123.7 (1.8)	46.0 (0.8) ***	347.9 (29.0) ^###^
PaCO_2 _(mmHg) (n = 6)	37.1 (1.2)	36.7 (1.1)	37.5 (0.8)
pH (n = 6)	7.42 (0.02)	7.42 (0.01)	7.40 (0.03)
[La] (mmol·l^-1^) (n = 6)	1.6 (0.4)	2.1 (0.7) ^+^	1.3 (0.3)
HR (bpm)	98 (23)	99 (23) ^+^	86 (16)
RPE (points)	6.0 (0.9)	5.8 (1.2)	5.2 (0.6)

Values are means (SD). ${\overset{•}{\text{V}}}_{E}$, minute ventilation; f_R _, breathing frequency; V_T _, tidal volume; Ti/Tt, ratio of inspiratory time to total respiratory cycle duration; P_ET _CO_2 _, end-tidal CO_2 _partial pressure; SpO_2 _, arterial O_2 _saturation; PaO_2 _, arterial oxygen partial pressure; PaCO_2 _, arterial carbon dioxide partial pressure; [La], blood lactate concentration; HR, heart rate; RPE, rate of perceived exertion. *** significantly different from normoxia and hyperoxia (*P *< 0.001), ^### ^significantly different from normoxia and hypoxia (*P *< 0.001), ^+ ^significantly different from hyperoxia (*P *< 0.05)

### Effect of hypoxia and hyperoxia on respiratory muscle twitch pressure at rest

After 10 min of hypoxic exposure at rest, there was no significant change in P_di,tw _and P_ga,tw _during cervical and thoracic stimulation, respectively (P_di,tw _: 31.9 ± 9.3 cmH_2 _O before vs 31.6 ± 9.1 cmH_2 _O after, n.s.; P_ga,tw _: 36.3 ± 6.5 cmH_2 _O before vs 34.5 ± 7.5 cmH_2 _O after, n.s.; n = 6). Similarly, after 10 min of hyperoxic exposure at rest, there was no significant change in P_di,tw _and P_ga,tw _(P_di,tw _: 28.2 ± 5.5 cmH_2 _O before vs 30.2 ± 7.1 cmH_2 _O after, n.s.; P_ga,tw _: 35.8 ± 13.3 cmH_2 _O before vs 35.7 ± 13.4 cmH_2 _O after, n.s.; n = 6).

### Respiratory muscle fatigue following hyperpnoea

P_di,tw _during cervical stimulation and P_ga,tw _during thoracic stimulation before the hyperpnoea test did not differ between conditions (Table 3).

**Table 3:** Absolute values of transdiaphragmatic twitch pressure during cervical magnetic stimulation and gastric twitch pressure during thoracic magnetic stimulation before and after the 15-min hyperpnoea test in normoxia, hypoxia and hyperoxia

	Normoxia	Hypoxia	Hyperoxia
P_di,tw _			
Before	30.6 (8.9)	31.4 (8.7)	31.7 (9.3)
Post 0	23.8 (6.6)	20.9 (7.3)	24.8 (6.4)
Post 30	27.5 (7.6)	26.6 (6.7)	28.7 (7.2)
P_ga,tw _			
Before	31.7 (7.1)	33.9 (7.9)	32.9 (9.2)
Post 0	25.9 (5.0)	24.6 (5.0)	27.0 (6.2)
Post 30	27.6 (6.2)	26.5 (8.0)	28.7 (8.3)

Values are means (SD). P_di,tw _, transdiaphragmatic twitch pressure during cervical magnetic stimulation; P_ga,tw _, gastric twitch pressure during thoracic magnetic stimulation; Before, before hyperpnoea; Post 0, immediately after hyperpnoea; Post 30, 30 min after hyperpnoea.

**Figure 1:**
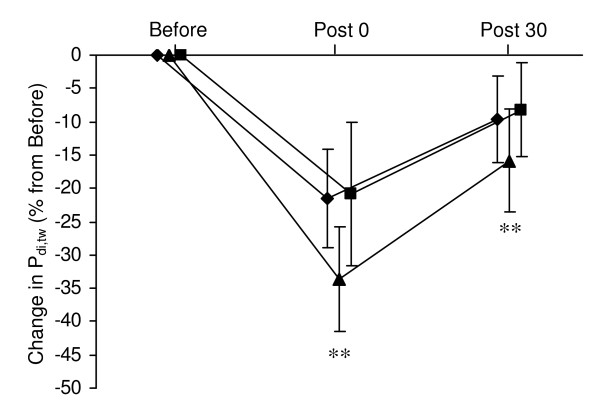
**Changes in transdiaphragmatic twitch pressure (P_di,tw _) during cervical magnetic stimulation immediately after and 30 min after the 15-min hyperpnoea test in normoxia (diamond), hypoxia (triangle) and hyperoxia (square)**. Values are mean ± SD. All values were significantly reduced immediately after and 30 min after hyperpnoea compared to before hyperpnoea. ** significantly different from normoxia and hyperoxia (*P *< 0.01).

**Figure 2:**
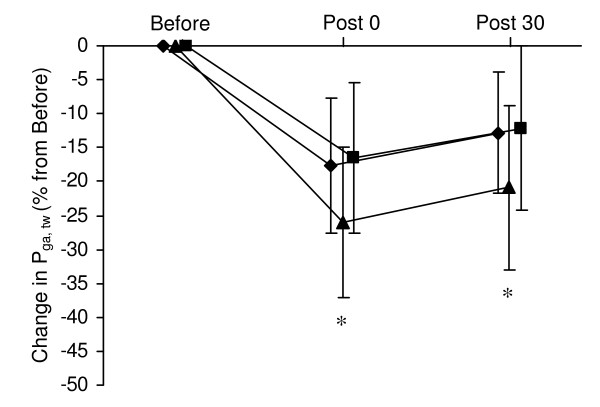
**Changes in gastric twitch pressure (P_ga,tw _) during thoracic magnetic stimulation immediately after and 30 min after the 15-min hyperpnoea test in normoxia (diamond), hypoxia (triangle) and hyperoxia (square)**. Values are mean ± SD. All values were significantly reduced immediately after and 30 min after hyperpnoea compared to before hyperpnoea. * significantly different from normoxia and hyperoxia (*P *< 0.05).

**Figure 3:**
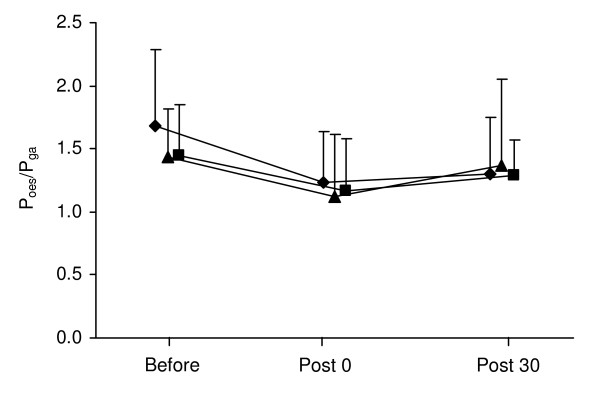
**Ratio of oesophageal and gastric twitch pressures (P_oes,tw _/P_ga,tw _) during cervical magnetic stimulation before, immediately after and 30 min after the 15-min hyperpnoea test in normoxia (diamond), hypoxia (triangle) and hyperoxia (square)**. Values are mean ± SD. All values were significantly reduced immediately after and 30 min after hyperpnoea compared to before hyperpnoea.

Changes P_di,tw _during cervical stimulation and P_ga,tw _during thoracic stimulation from before to after the hyperpnoea test are shown in Figures 1 and 2. In all three conditions, P_di,tw _and P_ga,tw _were significantly reduced immediately after hyperpnoea as well as after 30 min of recovery compared to before hyperpnoea. P_oes,tw _/P_ga,tw _ratio during cervical stimulation was significantly reduced after hyperpnoea in all three conditions (Figure 3).

The reduction in P_di,tw _during cervical stimulation as well as the reduction in P_ga,tw _during thoracic stimulation were significantly greater in hypoxia compared to normoxia and hyperoxia both immediately after hyperpnoea and after 30 min of recovery. Ten out of 12 subjects had greater P_di,tw _reduction in hypoxia versus normoxia and hyperoxia, while 9 out of 12 subjects had greater P_ga,tw _reduction in hypoxia versus normoxia and hyperoxia No significant difference in P_di,tw _or P_ga,tw _reductions was observed between the normoxic and hyperoxic conditions. Changes in P_oes,tw _/P_ga,tw _ratio during cervical stimulation did not differ between conditions. No test order effect was observed for twitch reduction after hyperpnoea (n.s.).

## Discussion

The present study evaluated for the first time the effect of arterial blood oxygenation on inspiratory and expiratory muscle fatigue induced by isolated voluntary hyperpnoea. The results showed that hypoxia (SpO_2 _= 80%) enhanced hyperpnoea-induced diaphragm and abdominal muscle fatigue compared to normoxic conditions, while hyperoxia (FiO_2 _= 0.60) had no significant effect on respiratory muscle fatigue. These findings provide objective evidence of significant hypoxic effects specifically on respiratory muscle fatigue as induced by hyperpnoea. They imply that hypoxia enhances hyperpnoea-induced respiratory muscle fatigue independently, at least in part, of its effects on the ventilatory response and the relative leg work intensity during whole-body exercise.

### Diaphragm fatigue following inspiratory resistive breathing in hypoxia

During inspiratory resistive breathing in dogs, diaphragm blood flow and O_2 _extraction was shown to increase exponentially [31]. In addition, hypoxia has been shown to be a potent diaphragm vasodilator [32,33]. Hence, blood flow to the diaphragm might be able to increase greatly under hypoxic conditions in order to maintain adequate O_2 _delivery, therefore avoiding fatigue exacerbation. Several studies in human assessed the effect of hypoxia on inspiratory muscle during inspiratory resistive breathing [17-19]. A FiO_2 _of 0.13 has been shown to decrease endurance time and to induce earlier shifts in the electromyogram frequency spectrum of the diaphragm compared to normoxic conditions [17,18], providing indirect evidences of greater inspiratory muscle fatigability in hypoxia. Conversely, Amaredes et al. [19] compared the reduction in maximal inspiratory mouth pressure during inspiratory muscle loading under normoxic, hypoxic and hyperoxic conditions and found similar amount of fatigue in all conditions. The limits of these studies are however i) to involve a specific form of loaded breathing substantially different from hyperpnoea and ii) to provide no objective measurements of muscle contractile fatigue. In addition, none of theses studies evaluated the effect of hypoxia on expiratory muscle fatigue.

### Diaphragm fatigue following whole body exercise in hypoxia

Several studies evaluated the effect of hypoxia on diaphragm fatigue by comparing the amount of fatigue observed after whole-body exercises performed under normoxic and hypoxic conditions [11-13]. These protocols, although reproducing conditions similar to those encountered during altitude exposure for example, make the evaluation of the specific effect of hypoxia on respiratory muscle fatigue difficult. Indeed, at similar exercise work output, hypoxia may increase diaphragm fatigue because of i) increased minute ventilation and therefore work of breathing [34] and/or ii) interaction between locomotor muscle work and respiratory muscles, i.e. concurrence for cardiac output [14,35] and/or iii) increased level of circulating metabolites (e.g. lactate) associated with locomotor muscles working at higher relative intensity in hypoxia. To avoid part of these confounding effects, Vogiatzis et al. [13] recently compared hypoxic and normoxic exercise at intensities that produced the same ventilatory level and therefore respiratory muscle work, which meant setting a lower leg work rate in hypoxia. Within these conditions, the authors found greater diaphragm fatigue in hypoxic conditions. However, although smaller in absolute value compared to normoxia, the leg work during hypoxia (when considered as a percentage of maximal hypoxic work rate) may still have had greater effect on respiratory muscle fatigue development than in normoxia by limiting blood flow available for the respiratory muscles [1] and/or by increasing levels of circulating metabolites (e.g. [La] [36]). Hence, from these studies, the specific effect of hypoxia on respiratory muscle fatigue remains to be clarified.

### Diaphragm and abdominal muscle fatigue following isolated voluntary hyperpnoea in hypoxia

To clarify the specific effects of reduced arterial blood oxygenation on both inspiratory and expiratory muscle fatigue during increased respiratory muscle work as induced by exercise for example, i.e. hyperpnoea, we used a standardized bout of hyperpnoea with measurements of P_di,tw _and P_ga,tw _during cervical and thoracic magnetic stimulation. The workload endured by the respiratory muscles is a critical determinant of the exercise-induced diaphragm fatigue since, for instance, unloading the respiratory muscles with the use of a proportional assist ventilator prevents diaphragm fatigue [37]. Therefore, in the present study, we aimed to compare hyperpnoea-induced respiratory muscle fatigue for identical ventilatory load by precisely matching minute ventilation and breathing pattern in all three conditions. Table 2 shows that subjects were able to precisely match there target ventilation and breathing pattern over the three test sessions. Accordingly, the strategy of matching ventilatory requirement between the tests allowed us to isolate the role of arterial hypoxemia per se on respiratory muscle fatigue.

Cervical and thoracic magnetic stimulation have been shown to be valuable tools for measuring diaphragm and abdominal muscle fatigue as induced by exercise-induced hyperpnoea for example [9,10,13,23]. We took particular care of potential confounding factors while using this technique, by confirming supramaximal stimulation on every test session, by checking lung volume (through continuous P_oes _recording) before each stimulation and by measuring fully potentiated twitches both before and after hyperpnoea (i.e. by performing maximal voluntary contractions before stimulations). Supramaximality of thoracic stimulation could not be confirmed however in three subjects as previously reported [9], but since these subjects showed data similar to the rest of the group, there were included in all analysis. The between-day coefficients of variation of P_di,tw _and P_ga,tw _confirmed the excellent reproducibility of these measurements. By using this technique, we were therefore able to specifically compare contractile diaphragm and abdominal muscle fatigue following normoxic, hypoxic and hyperoxic hyperpnoea.

Fifteen minutes of hyperpnoea in the present study induced significant amount of diaphragm and abdominal muscle fatigue similar to those previously reported following intensive whole body exercise [8,10,11,13]. Such a reduction in force response to single twitch immediately after fatiguing contractions remaining significant after 30 min of recovery is consistent with the presence of low frequency fatigue [22,38]. We found that hypoxia did not modify diaphragm and abdominal muscle strength at rest compared to normoxia, as previously observed for other muscles under baseline resting conditions while breathing hypoxic gas mixtures [15,39]. Conversely, hypoxia significantly exacerbated both diaphragm and abdominal muscle fatigue immediately after hyperpnoea by + 12% and + 9%, respectively, compared to normoxia. These results extended to the respiratory muscles the recent results from Katayama et al. [15] regarding locomotor muscles showing, with a similar methodological approach (i.e. with isolated muscle exercise and twitch force measurements), greater quadriceps muscle fatigability in hypoxia. Hence, despite high oxidative capacities and capillarisation [40], the diaphragm and the abdominal muscles fatigue to a greater extent during hyperpnoea when the arterial O_2 _content is reduced. The reduction in P_oes,tw _/P_ga,tw _ratio following hyperpnoea, indicating extra-diaphragmatic inspiratory muscle fatigue [29], was not significantly different between conditions. These results may indicate that hypoxia has a smaller impact on hyperpnoea-induced fatigue of the extra-diaphragmatic inspiratory muscles compared to the other respiratory muscles. This remains however to confirm since P_oes,tw _/P_ga,tw _ratio is an indirect index of extra-diaphragmatic inspiratory muscle fatigue. Hyperoxia on the other hand had no significant effect on hyperpnoea-induced diaphragm and abdominal muscle fatigue, suggesting that muscle O_2 _delivery during isolated normoxic hyperpnoea is already optimal.

Potential mechanisms for contractile fatigue involves the influence of intramuscular metabolite accumulation such as inorganic phosphate (Pi) and H^+ ^, which can provide inhibitory influences on force development and Ca^2 + ^sensitivity [41]. The higher [La] we observed in hypoxia compared to the other conditions (Table 2) may be associated with greater perturbations of muscle homeostasis. Muscle acidosis associated with hypoxia is usually proposed to be a possible mechanism for the reduction in muscle force production during hypoxia [42]. However, recent in vitro studies have questioned the deleterious role of H^+ ^in metabolic fatigue [43], and faster accumulation of Pi in hypoxia may be an alternative mechanisms able to accelerate contractile fatigue [44,45].

### Relevance for whole body exercise in hypoxia

These present findings are of relevance to better understand performance limitation under hypoxic conditions. Indeed, during whole body exercise in hypoxia, increased fatigability due to reduced O_2 _transport in addition to the increased work of breathing make the respiratory muscles particularly exposed to fatigue. Since respiratory muscle fatigue is now recognized as a significant contributor to whole body exercise performance [46], respiratory muscle fatigue may be therefore a major contributor to performance limitations in hypoxia. The potential systemic impact of increased respiratory muscle fatigue is illustrated in the present study by the higher HR response in hypoxia compared to normoxic and hyperoxic conditions (Table 2). Such a result may be the consequence of a greater cardiovascular response associated with a sympathetically mediated metaboreflex originating from the fatigued respiratory muscles [14]. A greater accumulation of lactic acid (as suggested by greater [La] in hypoxic condition) and other metabolic by-products within the respiratory muscles working in hypoxia may indeed stimulate type IV phrenic afferents [47], enhance sympathetic activity and eventually increase the cardiovascular response [48]. These results as well as there potential deleterious effects on exercise performance may apply to exercise at high altitude but also to exercise in hypoxemic patients, frequently combining reduced arterial O_2 _content and increased work of breathing due to elevated ventilatory demand and increased airway resistance as patients with chronic obstructive pulmonary disease.

In conclusion, the present study provides evidences for hypoxia-induced exacerbation of diaphragm and abdominal muscle contractile fatigue by using cervical and thoracic magnetic stimulation before and after a standardized bout of isolated voluntary hyperpnoea. Hyperoxia on the other hand did not reduce respiratory muscle fatigue following hyperpnoea. These results emphasize the potential role of respiratory muscle fatigue in exercise performance limitation under conditions coupling increased work of breathing and reduced O_2 _transport as during exercise in altitude or in hypoxemic patients.

## List of abbreviations

FiO_2 _: inspiratory oxygen fraction; FiCO_2 _: inspiratory carbon dioxide fraction; HR: heart rate; [La]: blood lactate concentration; MVV: maximal voluntary ventilation; P_oes _: oesophageal pressure; P_ga _: gastric pressure; P_di _: transdiaphragmatic pressure; P_di,tw _: transdiaphragmatic twitch pressure; P_ga,tw _: gastric twitch pressure; P_ET _CO_2 _: end-tidal partial CO_2 _pressure; SpO_2 _: arterial oxygen saturation

## Competing interests

The authors declare that they have no competing interests.

## Authors' contributions

SV and DB were involved in the conception and design of the experiment, data collection and analysis, interpretation of the data and drafting the manuscript. BW was involved in the conception of the experiment, data collection and interpretation of the data. All authors approved the final version of the present manuscript.
